# Concurrent Stenoocclusive Disease of Intracranial and Extracranial Arteries in a Patient with Polycythemia Vera

**DOI:** 10.1155/2012/151767

**Published:** 2012-05-29

**Authors:** Le H. Hua, Robert L. Dodd, Neil E. Schwartz

**Affiliations:** ^1^Department of Neurology and Neurological Sciences, Stanford University School of Medicine, 300 Pasteur Drive, Room A343, Stanford, CA 94305, USA; ^2^Department of Radiology and Neurosurgery, Stanford University School of Medicine, Stanford, CA 94305, USA

## Abstract

Moyamoya disease is a stenoocclusive disease involving the intracranial carotid and proximal middle cerebral arteries. There are rarely any additional extracranial stenoses occurring concurrently with moyamoya. The pathophysiology of moyamoya remains obscure, but hematologic disorders, notably sickle-cell anemia, have been associated in some cases. We describe the novel case of polycythemia vera associated with severe steno-occlusive disease of both intracranial and extracranial large arteries. A 47-year-old woman with polycythemia vera had multiple transient ischemic attacks, and noninvasive vessel imaging revealed steno-occlusive disease of bilateral supraclinoid internal carotid arteries with moyamoya-type collaterals, proximal left subclavian artery, right vertebral artery origin, bilateral renal arteries, superior mesenteric artery, and right common iliac artery. Laboratory workup for systemic vasculitis was negative. She required bilateral direct external carotid to internal carotid bypass procedures and percutaneous balloon angioplasty of her right VA origin stenosis. This case suggests that hematologic disorders can lead to vessel stenoses and occlusion. The pathophysiology may be due to a prothrombotic state leading to repeated endothelial injury, resultant intimal hyperplasia, and progressive steno-occlusion.

## 1. Case Report

A 47-year-old woman of Chinese ancestry with a history of polycythemia vera (PV) presented to our facility for evaluation of a carotid bruit. In the waiting room, she developed lightheadedness that persisted despite sitting down. Her blood pressure was 211/72 mmHg in the right arm and 125/75 mmHg in the left arm. Her lightheadedness resolved after ten minutes with supplemental oxygen. She felt “sweaty and clammy” in her right hand and numbness in her left hand. She had no other symptoms and had never had lightheadedness episodes previously. She was diagnosed with PV twelve years earlier by bone marrow biopsy after routine studies revealed an elevated hematocrit. She was noncompliant with hydroxyurea and aspirin and underwent yearly phlebotomies as needed; the most recent phlebotomy was six months previously.

**Figure 1:**
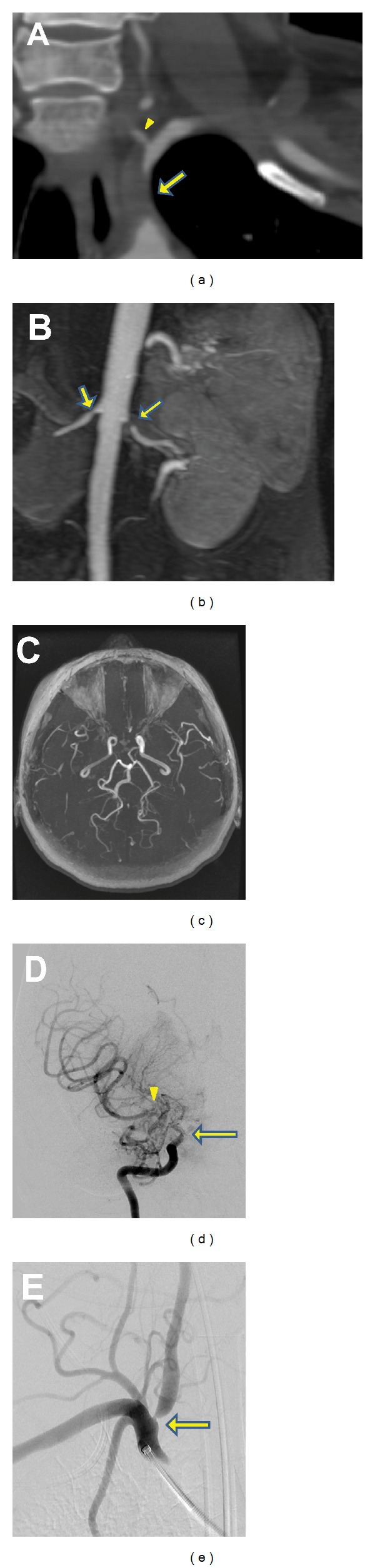
Multimodality imaging demonstrating stenoocclusive disease of intracranial and extracranial arteries. (a) CT angiogram chest demonstrating complete occlusion of left subclavian artery (arrow) with distal filling from left VA (arrowhead). Remainder of VA is out of plane of view, but patent distally. (b) MR Angiogram of abdomen demonstrating bilateral renal artery stenoses (arrows). (c) Time-of-flight MR angiogram of head showing absence of bilateral MCA vessels and decreased flow of both ICAs. The posterior circulation is relatively plethoric. (d) Conventional angiogram of the right ICA (AP projection) showing typical moyamoya appearance of intracranial occlusion of supraclinoid ICA (arrow) with reconstitution of the MCA via multiple small moyamoya collaterals (arrowhead). (e) Right subclavian digital subtraction angiogram demonstrating high-grade (>70%) right VA stenosis (arrow).

Her symptoms and differential blood pressure were concerning for subclavian steal phenomenon. This was confirmed on CT angiogram which demonstrated complete occlusion of the left subclavian artery proximal to the vertebral artery (VA) origin with a patent left vertebral and distal subclavian artery (Figure 1(a)). Interestingly, her CT angiogram also revealed severe stenosis at the origin of the right VA and stenoocclusive disease of the bilateral supraclinoid internal carotid arteries (ICA) with multiple small collaterals reconstituting the proximal middle cerebral (MCA) and anterior cerebral arteries. MR angiography confirmed the above arterial stenoses (Figure 1(c)), while MRI demonstrated multiple chronic watershed cerebral infarcts. Conventional angiogram also demonstrated bilateral supraclinoid ICA occlusions with moyamoya-type collaterals (Figure 1(d)), high-grade (70%) right VA stenosis (Figure 1(e)), and left subclavian artery occlusion. MR angiogram of her chest and abdomen demonstrated mild wall thickening of the aortic arch and abdominal aorta, 50% bilateral renal artery stenoses (Figure 1(b)), superior mesenteric artery stenosis, and right common iliac artery stenosis (not shown).

Laboratory analysis revealed normal leukocyte count of 8.7 K/*μ*L (normal 4–11) with normal differential, slightly elevated hemoglobin of 15.9 g/dL (normal 11.7–15.7) and elevated platelets of 660 K/*μ*L (normal 150–400). Antiphospholipid antibodies (lupus anticoagulant, anticardiolipin, and beta-2 glycoprotein) were absent. Other workup for systemic vasculitis was negative including c-reactive protein, erythrocyte sedimentation rate, rheumatoid factor, antineutrophilic antibody, antidouble stranded DNA, and anticytoplasmic neutrophil antibodies. Further testing revealed the presence of a Jak2 (V617F) mutation.

Her prior medications of aspirin 81 mg daily and hydroxyurea 1000 mg twice daily were restarted. Three days later, she developed transient right facial droop and right arm weakness that resolved spontaneously in a few hours. She was advised to undergo intracranial revascularization for her moyamoya syndrome. She was treated with bilateral direct external carotid to internal carotid bypass procedures. The patient continued to have episodes of lightheadedness, ongoing expressive aphasia, and left arm numbness. Some of this was attributed to her right VA stenosis as imaging after the revascularization procedures demonstrated patent bilateral carotid bypass grafts with improved blood flow. Six months later, she underwent percutaneous balloon angioplasty of her right VA origin stenosis with near complete resolution of her symptoms immediately following the procedure. Her extracranial stenoses remained stable on repeat imaging. Four months later, routine noninvasive follow-up imaging demonstrated restenosis of the right VA origin, and she began to develop mild dizziness, subtle gait ataxia, and occasional word finding difficulties. Repeat balloon angioplasty was again performed with immediate resolution of her symptoms. She continues to be on low-dose aspirin and hydroxyurea treatment for her PV with phlebotomies as needed.

## 2. Discussion

We report the first published case we are aware of: a patient with PV associated with severe multivessel stenoocclusive disease involving both the intracranial and extracranial circulations. Intracranially, the disease manifested in the typical angiographic changes associated with moyamoya syndrome. Extracranial involvement was seen in multiple large vessels off the aortic arch.

Moyamoya syndrome is a rare cause of stroke in young patients. It is due to progressive stenosis of the intracranial ICAs and their proximal branches with extensive collateralization; this latter finding produces the characteristic “puff of smoke” appearance on conventional angiogram (Figure 1(d)). The large vessel stenoocclusion is due to hyperplasia of smooth muscle cells and luminal thrombosis rather than atherosclerosis or inflammation [1]. The underlying etiology remains unknown, but genetic, acquired, and environmental factors have all been implicated [1, 2]. The cooccurrence of intracranial and extracranial vessel occlusion in moyamoya has not been previously reported in English.

Polycythemia vera (PV) is a myeloproliferative disorder characterized by excess erythrocytosis, but also leukocytosis, thrombocytosis, and bone marrow hypercellularity [3]. Although vascular complications occur in PV, including strokes, coronary artery disease, and erythromelalgia, the mechanism of thrombus formation is unclear [4]. Various mechanisms have been postulated including hypercoagulability from thrombocytosis, venous stasis, leukocytosis, and/or platelet dysfunction [4, 5]. In our patient, thrombosis does not appear to account for both the multiple large vessel stenoses extracranially and concurrent intracranial moyamoya syndrome. The cause of strokes and transient ischemic attacks in our patient was likely hemodynamic and due to decreased perfusion in the setting of cerebral vessel stenosis.

The most commonly reported hematologic condition associated with moyamoya is sickle-cell anemia (SCA) [1, 2]. Of the myeloproliferative disorders, there are only two case reports of moyamoya associated with essential thrombocythemia (ET) and none with PV [6, 7]. In SCA, moyamoya is thought to be due to progressive intimal and medial wall proliferation, endothelial irritation and edema due to repeated microvascular thrombosis [8]. Lazzaro et al. [6] suggest that ET may lead to moyamoya vascular changes in a similar fashion due to a prothrombotic state predisposing to endothelial injury and intimal hyperplasia, ultimately leading to progressive occlusion and thrombosis. They further suggest that hematologic disorders should affect all vascular beds, rather than to be restricted intracranially, and have pathologic support for this hypothesis with demonstration of intimal thickening of the superficial temporal artery. Our case further strengthens this hypothesis due to the presence of diffuse large extracranial stenoses.

It is unlikely that our case represents a systemic vasculitis, although we have no pathology to rule this out; inflammatory markers in our patient were negative. Further, the moyamoya changes would be most unusual in this setting. We suggest that PV patients who present with transient neurologic deficits undergo noninvasive angiography in addition to standard MRI to evaluate for the presence of stenoocclusive disease involving the cerebral circulation. This may identify patients who warrant further evaluation and intervention.
